# HyMoTrack: A Mobile AR Navigation System for Complex Indoor Environments

**DOI:** 10.3390/s16010017

**Published:** 2015-12-24

**Authors:** Georg Gerstweiler, Emanuel Vonach, Hannes Kaufmann

**Affiliations:** Institute of Software Technology and Interactive Systems, Vienna University of Technology, Favoritenstrasse 9-11-188/2, Vienna 1040, Austria; vonach@ims.tuwien.ac.at (E.V.); kaufmann@ims.tuwien.ac.at (H.K.)

**Keywords:** indoor tracking, navigation, localization, augmented reality, mobile

## Abstract

Navigating in unknown big indoor environments with static 2D maps is a challenge, especially when time is a critical factor. In order to provide a mobile assistant, capable of supporting people while navigating in indoor locations, an accurate and reliable localization system is required in almost every corner of the building. We present a solution to this problem through a hybrid tracking system specifically designed for complex indoor spaces, which runs on mobile devices like smartphones or tablets. The developed algorithm only uses the available sensors built into standard mobile devices, especially the inertial sensors and the RGB camera. The combination of multiple optical tracking technologies, such as 2D natural features and features of more complex three-dimensional structures guarantees the robustness of the system. All processing is done locally and no network connection is needed. State-of-the-art indoor tracking approaches use mainly radio-frequency signals like Wi-Fi or Bluetooth for localizing a user. In contrast to these approaches, the main advantage of the developed system is the capability of delivering a continuous 3D position and orientation of the mobile device with centimeter accuracy. This makes it usable for localization and 3D augmentation purposes, e.g. navigation tasks or location-based information visualization.

## 1. Introduction

Especially in big indoor environments such as airports, shopping malls, hospitals or exhibition halls, people are usually not familiar with the floor plan of the building and find it therefore difficult to get to a desired destination in time. For a mobile assistant, capable of supporting people while navigating in indoor locations, an accurate and reliable position of the user is required in almost every corner of the building. To provide a natural navigation experience a virtual navigation application, which is augmenting the real world video stream with location-based visual information would be very helpful. For displaying location-based information and the correct overlay on the mobile screen an exact location (position and orientation) must be computed in real-time on a mobile device. This enables the system to support position-dependent augmented reality (AR) applications not only for navigation but also for games, 3D inspection or education.

Global Positioning System (GPS) is frequently used in outdoor scenarios, but does not work reliably indoors. Competing state-of-the-art indoor localization approaches use so called beacons, which are devices distributed all over the building that broadcast a unique identifier. This technology, usually based on Bluetooth or other radio frequency (RF) technologies, only operates at a short distance and needs a complex infrastructure. Localization via WiFi requires a dense network of access points, which leads to high maintenance costs, but only provides positional accuracy of 1–3 m with only three degrees of freedom.

Indoor positioning on mobile devices is an actively researched topic [1,2]. Existing algorithms and technologies in this field do not provide the required centimeter tracking accuracy of the mobile device. The aforementioned indoor use cases pose specific requirements. Big closed tracking volumes have changing lightning situations, daylight and artificial light, seasonal decorations (e.g., at Christmas) and occlusions caused by moving objects or persons. A solution handling these challenges has not been published yet.

In order to precisely track the user for AR tasks only, computer vision methods (optical *i.e.*, visual feature tracking) in combination with other localization methods (usually inertial tracking) can be used. This has the advantage that no additional infrastructure is required due to the use of existing landmarks, thereby keeping the installation costs low. However, there are basic and applied research questions that hinder the use of existing solutions in the real world, e.g., how to increase robustness of existing algorithms for large scale tracking, how to reliably create a feature map of the whole indoor environment in a reasonable time, since especially public places like airports that cannot be shut down during standard operation times.

This paper presents the design and the development of a full mapping, tracking and visualization pipeline for large indoor environments. For this purpose a novel method of combining two different visual feature tracking algorithms was developed. In a first stage clearly recognizable area like advertisements, company logos or posters are used as visual descriptors for detecting the initial position and for continuous tracking. The second level of the hybrid solution is using an adapted visual simultaneous localization and mapping (SLAM) algorithm to achieve continuous tracking in almost all areas of the environment if none of the afore mentioned descriptors is available. As a fallback solution the data of the inertial sensor is used to bridge short distances where visual tracking does not work. To achieve this goal new concepts for all the individual components mentioned before had to be designed.

Mapping

- Capturing with an advanced computer vision (CV) camera system considering that the captured 3D maps will be used with a different camera setup on a mobile device;
- Fusion of 2D markers and 3D features under special conditions with respect to different scale values and storing the data into a global map structure for localization purposes;

Tracking

- Handling of multiple visual tracking solutions simultaneously on a mobile device, but still keeping computational effort low;
- Design of an intelligent fallback heuristic, which decides during tracking for the best alternative;
- Design of a predictive reinitialization procedure;

User Interaction

- Interface for exact placement of 3D location based content;
- Development of an AR navigation scenario;
- Testing the navigation scenario under real world conditions in a subsection of an airport.

The main contribution of the work at hand is a complete system, which enables user studies about user behavior, user interaction possibilities with AR content and other scientific research questions targeting large indoor AR enabled environments. Within this paper, we have developed a first scenario, which shows the potential for an end user AR application outside a desktop environment, the usual environment for visual SLAM approaches.

Section 2 of this article describes the differences to related work, followed by a description of the methodology in Section 3. Section 4 gives an overview of all developed software and hardware components needed for preparing a large indoor environment for an AR indoor navigation scenario. Technical details about the mapping and tracking process are described afterwards. We also present an evaluation scenario, which was specially developed for the Vienna International Airport in order to prove the applicability within a real world scenario. Finally, we summarize all collected results and experiences from all necessary steps containing capturing, mapping fusion and tracking.

## 2. Related Work

Indoor positioning systems capable of three degree of freedom (DOF) and six DOF pose tracking is an actively researched topic in different scientific communities, e.g., robotics, autonomous flight, automobiles, navigation, *etc.* A common research question is the estimation of an object’s or a user’s position within a large environment, with varying requirements on the tracking quality depending on the application domain. The proposed approach is based on two different visual tracking algorithms. The following sections will first describe approaches based on point features such as SLAM with different base technologies. In addition to that, related work dealing with 2D markers in big environments is discussed. In the final section of this chapter different approaches are mentioned, which cannot be used for indoor positioning on their own, but can extend the capabilities of a hybrid tracking system. Tracking technologies, designed for large areas like GPS, magnetic tracking or acoustic tracking are not included in the discussion, because of their low inherent accuracy and possible interferences, especially in big indoor environments.

### 2.1. Simultaneous Localization and Mapping

A large number of six DOF pose tracking approaches are based on the simultaneous localization and mapping (SLAM) concept. Originally indoor localization with SLAM emerged out of the robotics community [3] with the aim of driving robots autonomously through unknown environments without colliding with objects. With robots different high quality hardware such as laser scanners [3] or high performance cameras can be used. In addition to that high performance computers may be used which surpass the capacities of mobile devices considerably. For SLAM, different sensors and different kinds of signals e.g. RF signals and visual based methods (laser scanners, point features) may be used. The SLAM mapping process tries to extract spatial information (signal strength, 3D points…) of the real world environment in order to store it in a global reference map while at the same time keeping track of the agent’s position. When using smartphones as a base platform [1] low computational power, limited battery life and low quality sensors pose additional requirements on a SLAM algorithm. There are already different approaches and a broad number of concepts based on different hardware such as Bluetooth, WiFi or visual features. All of these technologies can be used for implementing a SLAM system for localization.

#### 2.1.1. WiFi SLAM

WiFi SLAM is frequently used for localization since many buildings are already equipped with numerous access points. For the mapping process the mobile device has to be moved through the whole environment in order to periodically measure the signal strength of all surrounding access points. The accuracy that can be achieved with WiFi SLAM is below one meter [4] in the best case. Some researchers have combined WiFi with Bluetooth beacons [5] in order to improve the accuracy in interesting areas. In any of these cases, the result of the tracking is a 2D position in a 2D map. Another topic, which is usually not discussed in these papers, is the update rate of the tracked position. Mobile hardware is usually restricted to scan the signal strength just a few times per second, which results in a very low refresh rate for new measurements. In addition to that, signals tend to be extremely noisy and signal strength highly depends on surrounding building structures and materials. For these reasons, WiFi SLAM on its own is not suitable for accurate interactive augmented reality applications. The main drawbacks of the systems are poor tracking quality, reliability and the missing degrees of freedom.

#### 2.1.2. Vision-Based SLAM

In order to reach the desired accuracy for AR applications, it is necessary to utilize vision based methods, which can provide real six degrees of freedom poses of a user’s device. Vision-based SLAM algorithms relying on point features are able to estimate a position as well as an orientation of a stereo setup. Spatial information in terms of point features like the Scale Invariant Feature Transform (SIFT) [6], the Features from Accelerated Segment Test (FAST) [7], the Oriented FAST and Rotated BRIEF (ORB) [8], *etc.* is extracted from sequent frames in order to simulate stereo vision. The baseline between two frames has to be high enough in order to estimate the distance of the features [9]. This data is stored in a global map representation. Over time, a certain area can be mapped and used for tracking.

In recent years, algorithms were developed by utilizing a monocular camera in desktop environments for localization and displaying augmented reality content. Parallel Tracking and Mapping (PTAM) [10,11] by Klein *et al.* allows one to map a complex 3D environment while at the same time estimating the camera pose. The limitation to small desktop environments is similar to the issue with other algorithms like the extended Kalman filter SLAM (EKF-SLAM) [12] or Fast-SLAM [13], mainly due to missing optimization algorithms dealing with loop closure, map size optimization and re-initialization. Furthermore, desktop grade computing power is required. These algorithms mostly suffer from a small drift over time and usually do not implement a method of detecting already visited places (loop closure), which often results in adding the same features multiple times. Other successful implementations were done by [14,15]. Since these algorithms were developed for monocular setups, several publications already tried to adapt them to mobile devices. Some approaches use panoramas [16,17] for matching the current view with the previously saved map, which works very well but only in the vicinity of the mapped panorama. Our approach aims for a continuous tracking over a distance of 100 meters and more.

The field-of-view of smartphones is usually very narrow around (60°–70°) and is therefore not ideal for narrow indoor environments. In addition, when dealing with wide spaces the reference maps are getting very large and acquiring a first position without prior knowledge can take several seconds by brute force search through a recorded map. To release the mobile device from intensive processing, Ventura *et al.* [18] published a client-server structure. The server is managing the global reference map, whereas the client unit is just building a local map, which is regularly exchanged for global localization. The advantage of this approach is the possibility of handling big environments, but a complex client-server structure including a continuous network connection is needed.

### 2.2. Marker-Based Tracking

Another possibility of creating a visual indoor localization system was developed by Wagner *et al.* [19]. They presented a solution based on fiducial markers, which were manually placed in an indoor environment. They achieved an overlay registration with augmented reality elements by using an early PDA like device with ARToolKit [20]. The advantage of using well-placed markers is unique identification within a very short time. However, equipping a public environment with fidicual markers is not reasonable, but using existing 2D textured surfaces with the approach of [21] could enhance the capabilities of a standard SLAM algorithm.

### 2.3. Complementary Tracking Technologies

Other technologies for extending visual tracking solutions can be integrated into a hybrid tracking system to either increase robustness or to serve as a backup solution. Beacons are small devices, which are distributed in the tracking area with high density. These beacons can use different signals in order to be detected by mobile devices. Usually Bluetooth [5], WiFi or even a combination of multiple signals [22] is used in order to estimate the approximate position of the user’s device. The accuracy depends on the amount of devices spread over the environment. As in other approaches, an accurate map of the beacons has to be generated. Usually algorithms estimating the position use the concept of Received Signal Strength Indicator (RSSI) [23] which delivers a more accurate position and does not just estimate the distance to the next beacon. It is not feasible to use this kind of technology within environments like airports, because of the high hardware and maintenance costs. In addition to that, it is not possible to detect the orientation of the device, which is essential for augmenting location-based information.

## 3. Experimental Section

### 3.1. Methodology

**Figure 1:**
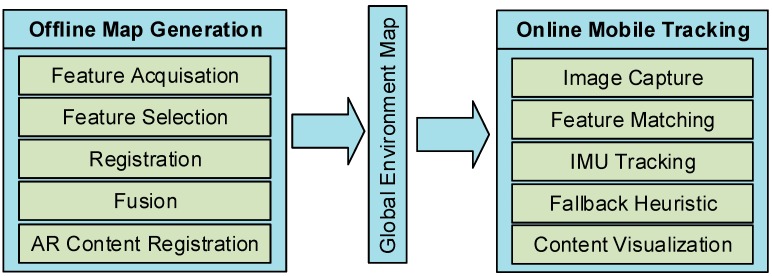
Developed packages for the HyMoTrack framework.

Our Hybrid Motion Tracking (HyMoTrack) framework contains multiple individual modules with novel concepts. It is capable of mapping a big indoor environment to place location-based AR content. Using a pre-recorded map, a mobile device is able to estimate a six DOF pose in real time while at the same time showing 3D content to the user. In order to lower computational power requirements while tracking and to focus on individual steps of the mapping process, the concept is divided into two parts. The first task is the mapping of the environment and the second task is about performing a real time tracking approach. The single steps of mapping and tracking are listed in Figure 1. They are briefly described in Section 3.1.1 and Section 3.1.2. Details are provided in Section 3.2.

The concept of the HyMoTrack framework is based on a hybrid tracking solution, which tries to exploit the capabilities of two different visual tracking technologies. Our approach targets locations such as airports, shopping malls, hospitals, *etc.*, which typically have controlled lighting situations during day and night times. Therefore a visual SLAM algorithm is very well suited for this application scenario. SLAM is used as the main tracking solution. We used the implementation by Metaio (http://www.metaio.com/), which is based on point features. It is capable of tracking constructional characteristics such as ledges, pillars or edges. The SLAM implementation of the Metaio SDK is similar to PTAM and runs on mobile devices. Since it is designed for small environments we implemented a novel algorithm for handling multiple small reference maps in order to extend the capabilities of the restricted Metaio framework to be used in big environments.

The indoor environments, which the presented framework is designed for, are already fitted with visual content like signs or company brands. These characteristics rarely change over time due to temporal decorations and can therefore be seen as permanent features in a constantly changing environment. Therefore, we use 2D feature markers as a secondary tracking solution to ensure that the most attractive areas to humans like signs and posters always result in a valid tracking position. For identifying and tracking these natural feature markers [21], the Metaio SDK is also used. By using only visual features for localization purposes, the presented concept does not require any additional hardware within the environment such as beacons, manually placed visual markers or any kind of Wi-Fi infrastructure.

#### 3.1.1. Mapping and Content Registration

In order to build a unique fingerprint of the environment multiple steps have to be performed (Figure 1, left part). The advantage of splitting the mapping of the environment from tracking is that a high performance notebook and professional computer vision camera can be used for mapping, which also results in a faster mapping process. In a first step, a map generation process has to be performed. This contains the feature acquisition, by capturing spatial information with the SLAM algorithm and distinctive 2D feature markers. This data is registered and fused into a global coordinate system. In addition to that, a 2D map of the environment is integrated into this reference map. This makes it possible to exactly place location based AR content within the environment. All the information, which is processed in these mapping and capturing steps is saved and transferred to a mobile device running the proposed tracking algorithm.

#### 3.1.2. Mobile Tracking Concept

Unlike the mapping process, tracking was developed for an Android smartphone, whereby all processing is done locally and no network connection is needed. The generated information of the environment is loaded on the mobile device. The tracking concept is responsible for managing the camera stream and feature matching with both visual tracking solutions. In order to achieve a robust estimation of the position and orientation, we developed a fallback heuristic. It is responsible of switching between the different tracking solutions. Therefore, it is possible to take the most reliable method at a certain situation and thereby minimizing the risk of losing the tracking while at the same time keeping the computational effort low.

If all tracking approaches fail due to any reason, we utilize the inertial sensors of the mobile device. This makes it possible to bypass short losses in the visual tracking system, thereby providing a seamless tracking experience. In addition to the tracking solution, we also present a concept for guiding people in indoor environments with AR content. The user is able to localize herself/himself by using an Android-based smartphone within a building with centimeter accuracy.

### 3.2. System Overview

The HyMoTrack framework consists of multiple interfaced subsystems responsible for capturing and mapping the environment, fusion of the used tracking technologies, placing 3D content and finally tracking a mobile device with the camera input. These modules are running on different platforms and devices, due to performance reasons. Since there are different steps needed until the global reference map is generated, the whole system is planned to work as automated as possible, but still needs some manual steps, mainly for copying metadata between the modules.

**Figure 2:**
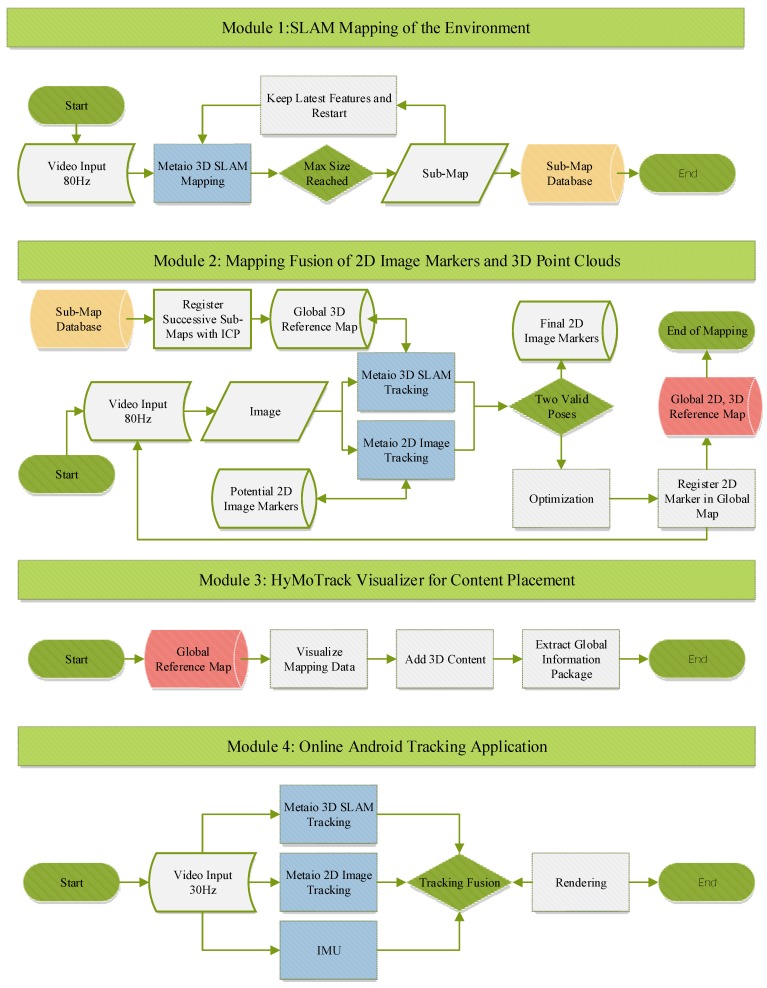
System workflow of the presented indoor tracking solution.

Figure 2 shows the designed workflow in detail. It contains all steps between capturing the environment and using a smartphone for tracking and visualizing AR content. In the project we utilize the functionality of the Metaio SDK since the main innovation is not the implementation of another SLAM algorithm, but showing the capabilities of the proposed hybrid mapping and tracking approach and how it can improve the usability of AR applications. We take advantage of parts of the Metaio SDK in six steps within the workflow (marked in blue in Figure 2). The SLAM approach is used for mapping only small chunks of the environment in a 3D point cloud to guarantee a good mapping quality. Furthermore, the tracking capabilities of the Metaio SLAM and Metaio image marker implementation is part of the fusion and the tracking process on the mobile device. In addition to that, the SDK is accessing the inertial sensor of the phone and therefore provides the functionality to predict the pose and the orientation for a specific number of frames when the visual tracking fails.

Within the workflow the modules one to three are responsible for creating a global reference map. The first component (module one) consist of an application, which is responsible for capturing 3D point clouds by using SLAM. In order to capture an environment in a reasonable time an external computer vision camera equipped with a wide-angle lens is connected to a notebook. For the mapping procedure, overlapping SLAM maps of the location of interest have to be captured. The maps including the video footage are saved on the notebook for registration purposes. In parallel, it is necessary to capture good textured 2D surfaces like signs or posters of the environment, which could have a good potential to be integrated as 2D feature markers into the global map. In addition to that, digital copies of posters or billboards can also be integrated into the list of feature markers.

The second module of the pipeline fuses the individual recorded information into a global coordinate system. In a first step the 3D maps have to be registered to each other. In a second step the image markers have to be registered with the previously recorded video footage. The result of the mapping procedure is an xml structured file describing the offset of the individual markers to the global origin.

After successful registration another application (module three) takes over. Therefore, the Unity 3D (https://unity3d.com/) editor was adapted to our needs. Unity 3D is a game engine with a flexible editor that we utilized as an interface for map editing. Thereby it is possible to load all markers and point clouds within one graphical user interface for evaluation and editing purposes. By overlaying a 2D map of the location, any 3D model can be exactly placed within the 3D environment for augmentation purposes.

The tracking application is running one thread for each tracking solution in parallel. Detecting the initial pose without any prior knowledge is implemented as well as a fallback heuristic, which decides which of the tracking technologies is suitable in the current situation. The mobile application is also handling a prediction model for loading suitable sub-maps and image markers at the right time in order to keep the time for re-initialization low. The following sections describe the designed workflow of each module presented in Figure 2.

### 3.3. Module 1: Mapping Large Environments

This section describes the workflow and implementation of the first module (Figure 2), which is responsible for mapping the environment with the chosen SLAM approach and capturing 2D textured surfaces for potential image markers.

#### 3.3.1. 3D Feature Mapping

In order to capture 3D spatial information of the environment, a Windows-based application was developed. A high performance notebook equipped with an Intel i7 processor was used in a mobile setup to capture the information. For capturing the environment, multiple cameras with different specifications regarding resolution, frame rate and field-of-view were tested. For the final mapping process a computer vision USB 3.0 camera (IDS-UI337, IDS Imaging Development Systems GmbH, Obersulm, Germany) with a resolution of 1024 by 1024 pixels and a maximum frame rate of 100 Hz was chosen. The squared sensor format had the advantage of detecting more important features on signs mounted on the ceiling. The high frame rate makes it possible to move faster through the environment without generating blurred images. A reasonable framerate of 80 Hz was chosen. In addition to that a global shutter ensures that the captured feature points are correctly aligned while moving the camera. The lens used for the camera had a focal length of 8 mm and therefore a wider field-of-view than usual smartphone cameras. With this setup we were able to capture images within a field-of-view of about 85 degrees in the horizontal and the same in the vertical angle of view. This has the advantage of capturing more features with one frame compared to usual lenses.

**Figure 3:**
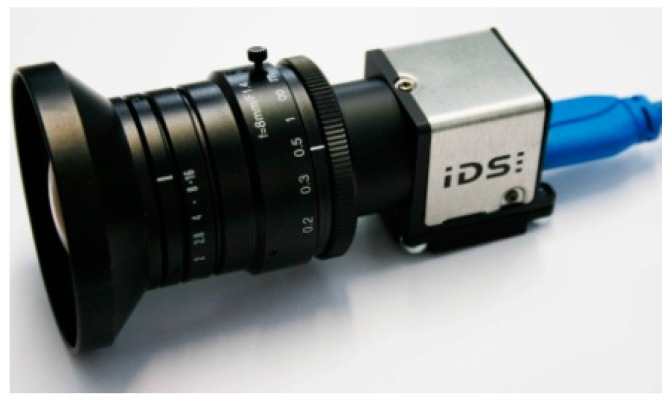
IDS camera UI337, resolution: 1024 by 1024 pixels at 100 Hz.

**Figure 4:**
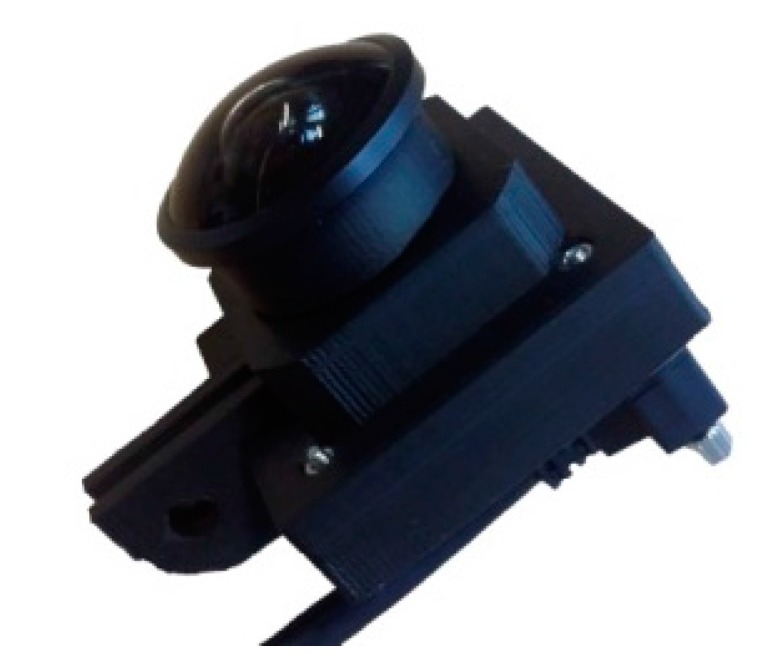
Low-cost IDS camera UI-3251LE-M-GL, resolution: 1600 by 1200 pixels at 60 Hz.

The intrinsic calibration of the camera was done with the Matlab Calibration Toolbox based on [24] in order to distort the image according to the lens distortion parameters. This makes it possible to use the captured map with other calibrated hardware setups like smartphones. Figure 3 shows the used camera for the setup. Since this camera setup has a high price of EUR 5000, later on in the project we discovered and tested a cheaper alternative camera-lens combination with similar specifications available for EUR 400. We were able to achieve comparable map quality with the IDS-UI_3251LE_M_GL camera shown in Figure 4.

As a base for our work the SLAM implementation of the Metaio SDK was used. To achieve fast loading times on the mobile devices only small manageable sub-maps were recorded. Since monocular SLAM algorithms are not able to measure the baseline between two keyframes, the scale of a recorded map is only estimated. The proposed approach however requires not only the same scale for every map recorded, but also the real world scale. Two features in the point cloud have to have the same distance as the corresponding points in the real world. This is necessary because the 2D image markers used in the project have fixed measurements (see next chapter) and will be registered within the point cloud. To ensure a correct registration we had to define the scale of the sub-maps with an artificially placed fiducial marker. Before recording the first map, a 2D fiducial marker of fixed size (usually with an edge length of 25 cm) was placed in the environment.

The Metaio SLAM algorithm is able to start with the tracking of the 2D marker. It then expands the tracking with the SLAM approach thereby using the image marker features within the SLAM map, thereby defining the real world scale. This approach guarantees the scale for the first map. Another advantage of this method is, that the usual initial stereo matching step is obsolete, which can be challenging in certain conditions. In this way the mapping process can start as soon as the marker is detected. The developed mapping application occasionally saves the map in order to keep the sub-maps small. Several tests showed that point clouds with more than 2000 features tend to drift dramatically in big environments. For that reason, the maximum number of features in one captured sub-map was limited to 2000. Another advantage of small maps is the size. One map can be calculated with about 500 KB per map, which has a significant effect on loading times. The mapping process is a continuous process in our approach. Every time a map is saved all features of the previously saved map are deleted and the currently saved sub-map is loaded again into the mapping process. This ensures that the sub-maps are next to each other and share only a small part of the features. Furthermore, by using preceding features in new sub-maps it is guaranteed that the scale of the map stays the same over time. During this whole process the video footage is captured for later use in order to register natural feature markers in the global reference map.

**Figure 5:**
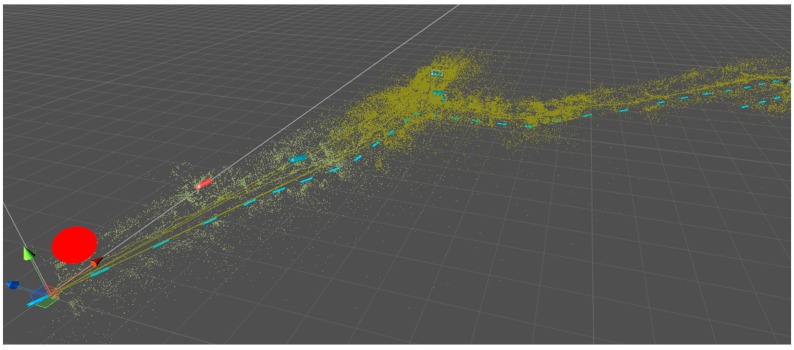
Visualization of mapped point features of a region covering a distance of 150 meters. The red point correlates to the starting position of the mapping process, whereas the blue dashed line shows the path to a target area.

The result of a mapping process can be seen in Figure 5. Within the 150 m rage 10 maps were saved while walking along the path in one direction. It can be clearly seen that the density of the features is varying. As a consequence, the size of the area covered by a sub-maps varies according to the scenery. Long hallways like in the lower left corner of the image are represented through sparse point clouds in contrast to environments like the shopping area (middle of the image), where many visual features are available and one map only covers a few meters of the path.

#### 3.3.2. 2D Image Markers

Public places are usually fitted with 2D textured surfaces like signs, advertisements or paintings. These areas contain “good” textures that provide many features for tracking. SLAM algorithm usually try to capture features, which are equally distributed over the captured frame in order not to add too many features to the map. Because of this, the SLAM mapping process adds too less features in such important textured surfaces in order to start tracking from a close distance. For that reason, we integrated tracking of 2D image markers with the Metaio implementation of marker less image tracking to increase robustness of our solution. This allows the framework to add almost any rectangular textured surface as a marker, although not every texture is suitable, due to low contrasts or missing corners in the image. The image markers also have the function of detecting the initial position of the device, if no prior knowledge is available. In contrast to 3D point features, the image markers have to be pre-processed manually. In order to get a digital representation of the desired areas it is necessary to take a picture at a certain quality. In order to use the right scale, the size of the markers must be measured. These steps are only relevant if it is not possible to get access to digital originals. In the future the authors plan to automate these steps by scanning the environment with a combination of an RGB and depth camera in order to detect good textured planar surfaces. With the depth camera it would also be possible to measure the size of the detected area.

**Figure 6:**
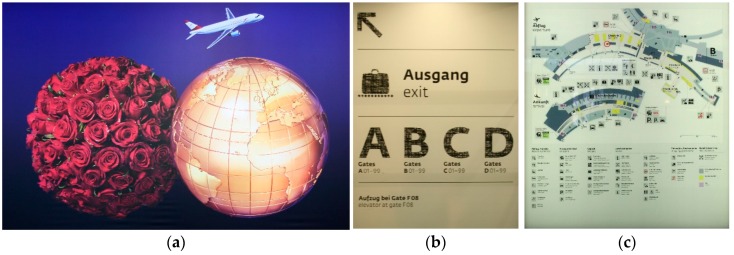
Examples of textured surfaces used as 2D image markers. (**a**) Advertisement; (**b**) Destination board; (**c**) General map.

Figure 6 shows some examples of selected textures, which were used as feature markers in the testing environment. Especially in big indoor environments similar or even the same advertisements usually appear multiple times. Therefore, for every representation the number of occurrence is noted and we use only one digital representation repeatedly.

### 3.4. Module 2: Fusion

The mapping fusion process described as module 2 in Figure 2, describing the framework, is an important step within the whole process. The fusion of the multiple tracking technologies has to be done very carefully in order to guarantee a useable tracking performance.

For this process a Windows application was developed. It is accessing the SLAM maps and the markerless image tracking technology of the Metaio framework. In a first step, all captured SLAM maps are registered within one global coordinate system. Two alternatives have been implemented to connect two successive maps. The first uses an iterative closest point (ICP) [25] algorithm to get the relation between two successive point clouds. The ICP from the point cloud library (PCL): (http://docs.pointclouds.org/trunk/classpcl_1_1_iterative_closest_point.html) is used to minimize the distance between two point clouds. The result of this process is converted into translation and rotation vectors, describing the offset between the two local coordinate systems. If this is not successful, because of a gap in the mapping process or having an insufficient number of matching features, the saved video footage is used to extend the first map in order to add more features to increase the probability of finding a reliable match with the ICP algorithm. The sub-maps are not fused together into one single map file, but the information about the offsets between each individual map is saved in order to be able to visualize the point cloud. Figure 5 shows the result of a globally fused reference map.

For adding image markers to the created global reference map structure, it is necessary to build a database of all captured images. In an XML file all necessary information is collected containing size, image path, ID and number of repetitions. The developed algorithm to register the markers within the global map uses the captured video footage of the mapping process. Every frame of the captured video passes through two instances. In the first instance, the 3D SLAM is tracking the position of the camera according to the fused sub-maps. In order to guarantee a continuous tracking the currently active SLAM map is extendable with a maximum of 500 features, after that the adjacent map is loaded for further tracking. The current image is then handed over to the 2D image marker tracking instance, which is scanning the image for any relevant image markers of the database. The used hardware allowed the scanning of 100 markers per second without dropping below 30 fps during the fusion process. If an image marker is found the pose as well as the tracking quality is monitored over time. The final position of the marker is calculated according to the average pose of the marker and weighted by the monitored tracking quality over time in order to estimate a feasible global pose. In case a poster is changed in the mapped environment, it is possible to just exchange the image, thereby keeping the metadata like size and position and keeping the map up-to-date.

As a result a well-structured XML file is generated. It contains the pose in relation to the global coordinate system of each registered marker and each SLAM map. The package, which is containing the information for the tracking device is including the XML file, the raw 2D markers and the point clouds.

### 3.5. Module 3: Viewer

Before loading the reference map on a mobile device for tracking, the data is visualized in the content viewer application. For this purpose, we developed an interface with the editor of the Unity (https://unity3d.com/) game engine. It has three main purposes: visualization and editing of mapped data and placing 3D objects for interactive AR content.

**Figure 7:**
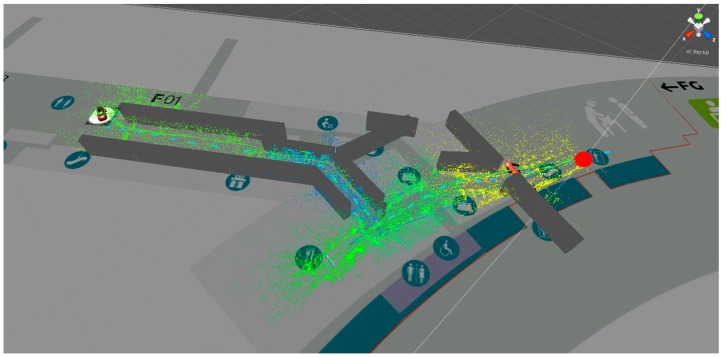
Screenshot of the content viewer.

In a first step, the metadata of the mapping process is loaded and all content containing the SLAM point clouds as well as the 2D image markers are visualized within a global coordinate framework. A first evaluation of the mapping procedure can be done with this first step. Figure 7 shows a screenshot of the application. Furthermore, in case the location has changed it is possible to edit or replace different markers within the environment, for example due to an exchanged advertisement or because a marker was removed.

In order to demonstrate the usability of such a system the content viewer was extended for adding AR content. Because it is hard to relate a position within a point cloud with a real world position a simplified version of a 2D map of the environment can be registered within the mapped data. For the testing location a starting point and an endpoint can be defined. We integrated a path planning algorithm [26,27], which calculates a path between these two points. For the AR navigation scenario, arrows were placed along the path. The path will be shown to the user within the camera stream. Since these are overlaid information, it is also necessary to deal with occlusions to present a realistic and understandable visualization of the path. The virtual scene contains all arrows. As soon as the tracking starts, the whole path will be visible to the user. Due to walls this can lead to a wrong registration in the real world. Because of that, simplified walls are placed according to the 2D map representation, which are blocking arrows that are not in the direct line of sight of the user.

### 3.6. Module 4: Tracking in Large Environments

All the information about the global reference map and the 3D models is transferred to the mobile tracking application, which is implemented for an Android device. As a testing platform a Samsung Note 2014 Edition equipped with an Exynos 5 Octa processor (4 × 1.90 GHz) was used. The main advantage of the presented solution is the fact, that two major tracking concepts are available to estimate the position of the user respectively the smartphone. To achieve this two threads are running in parallel, each one responsible for one tracking method. The tracking procedure is based on a multi-level approach, which starts with the detection of the initial position.

First the video stream is searched for a match with the 2D image marker database. For this purpose, the complete set of 2D markers is loaded into the tracking thread. Each frame is only scanned for a package of ten image markers in order to prevent a drop of the frame rate below 20 fps. A search for an initial position with the available SLAM maps would take too long, because of a loading time between 0.1 to 0.5 s per map. As soon as enough markers have been identified to pinpoint the location, both tracking threads are updated with a set of surrounding markers and maps in order to keep loading times at a minimum. The image marker thread is provided with 20 images whereas the SLAM thread holds five maps of the surrounding environment.

**Figure 8:**
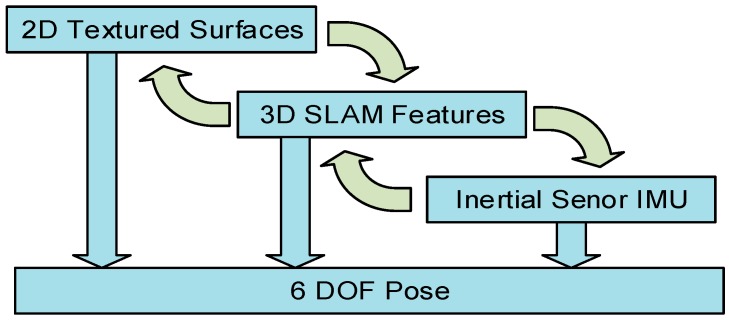
The waterfall model shows the combination of the different tracking methods.

As long as the detected image marker delivers a pose, the SLAM thread is running in parallel searching for a match. In general SLAM tracking is preferred, which means as soon as a valid position can be estimated, the 2D image tracking is set on pause. The currently active map is enabled to be extended for 500 features in order to guarantee a more solid tracking. This extended map is not saved to the database, but used as long as the device is in the near vicinity of that map. This ensures that the reference map is not degenerating over time. While the user is walking through the environment, the feature-sets loaded in both threads are updated according to the current position. Since loading the features takes some time, this procedure is alternately done in one thread while the other one is still able to deliver a pose. During the tracking process a fallback heuristic (see Figure 8) is handling the switching between the two visual approaches and the estimation of the inertial sensor. It is also responsible for activating and pausing the tracking technologies at the right time, since every running instance is also consuming additional computing power. For estimating the first position, the SLAM thread is deactivated and is waiting for the 2D marker thread to find a first position. Due to the multiple occurrence of the same 2D markers in several places in the environment, the SLAM approach is started as soon as the 2D tracking thread limits the number of possible areas down to two. This can be done, because during the capturing process the occurrence of each marker type is saven in the 2D marker database. As soon as this happens the SLAM algorithm is activated and loaded with maps from both possible areas. Both threads are then searching for a correct match. Depending on which thread is successful in finding the correct position, different choices are made. If the 2D marker thread is successful, the SLAM approach is loaded with maps that correspond to the found position. If the SLAM approach is successful first, the 2D thread will be deactivated.

In an ideal case, the SLAM thread is now able to provide a tracking position. Since we know the camera pose, the 2D thread will be activated in two situations. First, if the fallback heuristic detects that the camera is pointed towards a virtual 2D marker. The second reason for activation is if the tracking quality gets low, which is reported by the Metaio SDK in an interval [0, 1].

The fallback heristic is also able to set specific values like the number of frames to control the inertial sensor. The IMU data is used to predict the pose and the orientation for a specific number of frames. Since a high drift is expected the tracking data from the IMU is only used for a duration of 30 frames, which usually correlates to a duration of one second. This is enough time to bridge the gap between switching of two maps.

**Figure 9:**
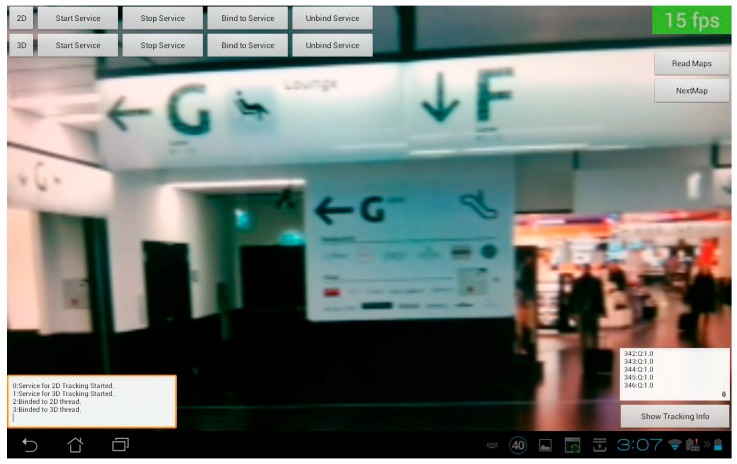
Screenshot of the tracking application.

**Figure 10:**
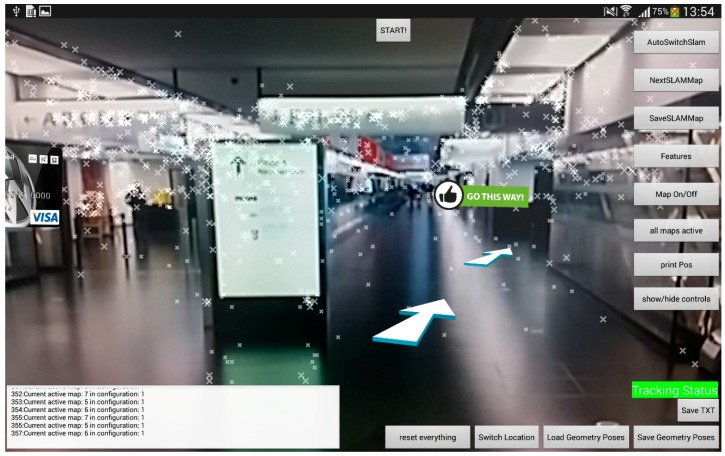
Tracking application including point features and path visualization.

Figure 9 and Figure 10 show two screenshots of the developed application. In Figure 9 no tracking is available at the moment and both threads are searching for a match. For this reason, the computational effort is high and the frame rate drops to 15 fps. Figure 10 on the other hand shows a valid tracking with the SLAM approach including the point features of the map and the visualization of the path.

### 3.7. Evaluation Scenario

Since the interest in an accurate indoor navigation system is high, the Vienna International Airport gave us access to a security area for our evaluation. It was possible to gain a lot of experience by testing the system under authentic conditions with real world challenges. Together with staff members an especially neuralgic location was chosen, where passengers usually have orientation problems. The selected area contains a path of about 150 meters, which is equivalent to a region of about 2000 m², where the passengers have to decide two times for the right direction.

Figure 9 shows the starting point right after the security check where people have to choose between two paths. The left way is just a small passage in contrast to the bright and visually attractive direction through the shopping area. In this environment there are several tracking challenges. It starts at a long wide hallway with partly daylight, but less features and some signs. Next the path leads through the previously mentioned shopping area, which is full of natural features and potential 2D image markers. This area is of special interest, because of a constantly changing environment, due to a dynamic product range. Finally, a long hallway is leading to 33 different gates with a high amount of similarities. The path ends at a specific gate along this corridor.

The interface of the tracking application is reduced to the camera stream and the AR content. The path that is visualized for the user consists of arrows, which are places approximately five meters apart. In addition to that a sign is telling the user when the desired destination is reached.

## 4. Results and Discussion

This section is discussing the evaluation of the system, which was done on the airport. In addition to that, the experiences of setting up a tracking system within a public environment will be integrated into the discussion. At the beginning, the experiences of the mapping step are described, followed by the tracking step. Furthermore, the technical details are listed. The paper concludes with a report about the experience of the people, who used the system for navigation through the airport.

### 4.1. Mapping

There are special challenges when mapping a public building like an airport. The mapping of point features should be done with lightning situations similar to the tracking conditions. Because the airport cannot close off the environment for a longer time, the mapping process has to be done during operation. Since the reference map has to be as perfect as possible, adding non-permanent features has to be avoided during the mapping procedure. At this point, the concept of mapping an environment with multiple small maps helps, because it makes it easy to react to sudden changes like the appearance of a crowd. Dynamic features which are not occluding important areas of the environment are usually not a problem when using a SLAM algorithm, because usually the RANSAC [28,29] or a similar algorithm is removing these features as outliers. For the airport scenario especially features on the ceiling like signs or special structures were very important in order to keep the tracking alive. For the whole path in one direction only 4,5 MB of point cloud data was captured within ten maps. This makes it possible to save all relevant data for tracking directly on the mobile device.

Capturing and preparing the 2D markers is a highly manual step at that moment. For the testing environment 23 image markers could be found, which were able to be tracked with sufficient quality. It took approximately four hours in order to collect all necessary map data (SLAM maps as well as 2D image marker) of the target area. However a large part of the time on sight was spent with waiting for big groups of passengers to pass.

### 4.2. Tracking

Tracking within the airport is a big challenge, due to very different circumstances like daylight, artificial light and long corridors with similar structures. Our solution could handle all of these situations quite well, except a large window environment where outside features were registered in the map, but couldn’t be detected during the tracking process. The initialization pose without any previous knowledge of the position could be found usually within three seconds, depending on the viewing direction of the camera, which was usually containing at least one 2D image marker. It was also tested to get the initial pose only by using the SLAM thread. In this case, the time for initialization highly depends on the number of maps in the database, since the loading time is much higher. For small indoor environments SLAM maps can be preferred for detecting an arbitrary position since any viewing direction should contain sparse point features. During our tests a reasonable set of five small SLAM maps for initialization of a position was always found within five seconds, whereas the loading consumes most of the time. The fast matching of SLAM maps can also be proved with the re-initialization when tracking is lost, by looking on the floor or covering the lens. In this situation mainly the SLAM algorithm finds the position within a couple of frames after the camera is pointed up again. When searching with a SLAM map it turned out that at least five frames are needed in order to find a position within a mapped environment. During tracking tests it was discovered that reoccurring structures are only a problem if they look exactly the same and are close to each other. There are two facts which keep the possibility of a mismatch low: First the implementation of using the combination of two tracking solutions following the path of the user. Second the fact that only the surrounding maps are kept active during the tracking process.

2D feature markers showed a good performance especially in situations where the camera was very close to certain objects, since the SLAM map usually does not contain enough point features to initialize at short distances. The small field-of-view of smartphone cameras did not have a positive effect on the tracking, but within the airport scenario the hallways were big enough to be able to overcome this problem.

An important part in tracking applications is the accuracy of the method used. Since the proposed approach is using different technologies during the tracking process, no global quality factor can be determined. The main advantage of using small SLAM maps is that the accuracy is independent of the size of the mapped environment. Every new loaded sub-map for tracking starts with an optimal pose. Therefore, the accuracy of our approach is dependent on the quality of the SLAM algorithm, which is usually in the magnitude of millimeters. The exact error could not be measured within the tested environment. Within such big environments it is almost impossible to setup a reference tracking system in the mapped area in order to generate ground truth values, due to costs and size. Unfortunately in this case we had no way to confirm the absolute accuracy of the system over the whole mapped area. However, since the mapping process is split up into multiple sub-maps, the tracking quality is equal to the quality of the Metaio SLAM implementation, which is generally below centimetre accuracy.

Defining the quality of the 2D markers is even harder, since the 2D textured areas are of different quality concerning the detected features, size and position in the room. However, if the viewing angle is below 30 degrees of the center, tests showed a jitter free visualization of the visual 3D content. In general, both visual approaches are qualified in order to augment 3D content at almost any position within the mapped environment.

### 4.3. Testing

An application for users, especially when dealing with interactive AR content, has to be highly responsive with real-time performance. In the worst case, when both tracking technologies were searching for a match, the frame rate drops to 15 fps, but as soon as a tracking pose is available, between 20 and 25 fps can be reached, which is high enough for real-time interaction.

**Figure 11:**
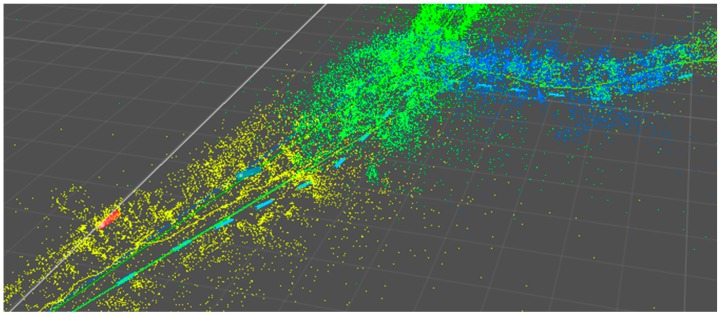
Point cloud of the mapped area on the airport. Lines show the distance traveled. Red (stop sign) and blue (path) rectangles are the AR content visualized for the user during a tracking session.

With the presented tracking solution it was possible to walk along the described path with continuous tracking. Figure 11 shows part of the map with the walked path, whereas the color of each point represents the ownership of the corresponding recorded sub-map. Since the maps have to be switched from time to time, a noticeable but not disturbing jitter occurs.

A direct comparison between two tracking runs is hard to make, since every walkthrough of a person has different conditions like a different path and different amount of people passing by and as already mentioned before no ground truth is available at the airport. In order to evaluate the robustness of the solution we performed a walkthrough along the mapped environment four weeks after the mapping process was done. Within this timeframe several parts of the airport changed the appearance. A wall directly facing the camera path was redesigned, the shops partly changed the product line and some smaller construction work was in process at the side of the hallway. Due to the high number of features within the map and the 2D image markers, the tracking was still working all along the path. Similar experiences were made when bigger groups of people were entering the field-of-view of the camera. To our experience, almost 50% of the image could be covered without resulting in loss of tracking, due to the features placed on the ceiling of the airport, like signs or brand logos above the shops. Considering that 100 feature matches are enough in order to estimate a position in a feature rich environment like in shopping areas, where there could be thousands of trackable features, extensive changes in the environment would have to be performed to prevent the tracking from working. In addition to that, as soon as the mobile application has produced a reliable position, the map is extended and the changed environment is also used for positional tracking in this situation.

**Figure 12:**
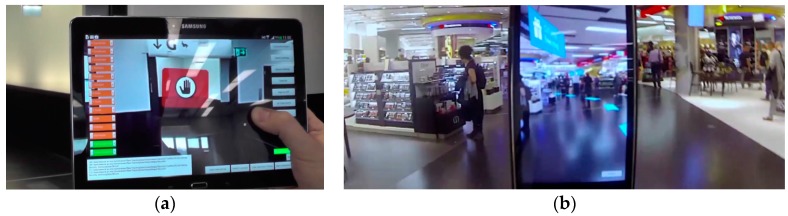
(**a**) The user was looking at the wrong passage indicated by the red sign; (**b**) Example of visualizing a path calculated especially for the user to find the way to the gate.

The tracking tests were done with a smartphone and a tablet. Figure 12a shows a situation where a route is blocked through a virtual sign for the user. Figure 12b on the other hand shows a path visualized through the shopping area with a number of arrows while considering the occlusion through walls. During the tests on the airport ten people were using the system. They were led over the whole distance of 150 m to the gate. Within these observed user tests two ways of using the system could be identified, whereas the majority was using both approaches. First, the device was used all the time to explore the AR environment and therefore a continuous tracking was needed. Later on the path people tend to look away from the screen, pointing the camera away from the walking direction for example towards the floor, thereby loosing tracking. Only after a while, they demand for tracking again in order to get new information on the path.

## 5. Conclusions and Future Work

In this paper, we have proposed a complete framework specially designed for an augmented reality indoor application. The mobile real-time implementation of the localization and visualization modules enables non-professional users to walk through an unknown environment while being navigated with a mobile device. The framework builds upon multiple novel concepts, which were developed and tested:

1. A mapping pipeline based on small SLAM maps with a wide angle camera
2. A high quality offline fusion module for SLAM maps and 2D feature markers
3. An interface for generating paths and placing 3D content for AR visualization
4. A mobile hybrid tracking concept with a fallback heuristic
5. An independent mobile tracking solution at real-time frame rates
6. Low hardware costs for big indoor environments

Compared to previous methods for tracking the HyMoTrack framework is a complete tool for setting up an environment for indoor localization and AR visualization like path planning or location-based information. We have demonstrated the usability of the tracking solution for non-professionals within a realistic airport scenario. The system showed a robust performance despite the challenging influences of a real environment like occlusions from passing people, different light situations or different moving speeds of users. The user tests also showed the intuitive usability of an AR based navigation system. Future work will be done dealing with a higher degree of automation especially for automated capturing and evaluation of 2D feature markers. The main contribution of the work at hand is a complete system, which enables user studies about user behavior, user interaction possibilities with AR content and other scientific research questions targeting large indoor AR enabled environments.
